# A critical assessment of Traditional Chinese Medicine databases as a source for drug discovery

**DOI:** 10.3389/fphar.2024.1303693

**Published:** 2024-04-26

**Authors:** Yinyin Wang, Minxia Liu, Mohieddin Jafari, Jing Tang

**Affiliations:** ^1^ School of Traditional Chinese Pharmacy, China Pharmaceutical University, Nanjing, China; ^2^ Faculty of Life Science, Anhui Medical University, Hefei, China; ^3^ Department Biochemistry and Developmental Biology, University of Helsinki, Helsinki, Finland; ^4^ Research Program in Systems Oncology, Faculty of Medicine, University of Helsinki, Helsinki, Finland

**Keywords:** Traditional Chinese Medicine, TCM databases, network pharmacology, mechanisms of action, drug discovery

## Abstract

Traditional Chinese Medicine (TCM) has been used for thousands of years to treat human diseases. Recently, many databases have been devoted to studying TCM pharmacology. Most of these databases include information about the active ingredients of TCM herbs and their disease indications. These databases enable researchers to interrogate the mechanisms of action of TCM systematically. However, there is a need for comparative studies of these databases, as they are derived from various resources with different data processing methods. In this review, we provide a comprehensive analysis of the existing TCM databases. We found that the information complements each other by comparing herbs, ingredients, and herb-ingredient pairs in these databases. Therefore, data harmonization is vital to use all the available information fully. Moreover, different TCM databases may contain various annotation types for herbs or ingredients, notably for the chemical structure of ingredients, making it challenging to integrate data from them. We also highlight the latest TCM databases on symptoms or gene expressions, suggesting that using multi-omics data and advanced bioinformatics approaches may provide new insights for drug discovery in TCM. In summary, such a comparative study would help improve the understanding of data complexity that may ultimately motivate more efficient and more standardized strategies towards the digitalization of TCM.

## 1 Introduction

TCM has not only played a crucial role in the treatment and prevention of disease in ancient times but also is used as a valuable source of natural products in modern drug discovery (Atanasov et al., 2021; Ngo et al., 2013). At present, there are more than 8,000 TCM components in total, which have been reported to have various pharmacological effects (Wangkheirakpam et al., 2018), especially for complex diseases (Yao et al., 2021), such as obesity (Vermaak et al., 2011), nonalcoholic fatty liver disease (Yan et al., 2020), cancer (Wang et al., 2021), and diabetes (Tong et al., 2012). TCM herbs as plant-based substances for medicinal purposes typically refer to the leaves, flowers, stems, seeds, or roots of plants that may induce potential health benefits. They can be used either naturally or as preparations. TCM herbs, as one particular type of natural products, have become increasingly popular in drug discovery in recent years. There are 3,322 clinical trials registered during 1999–2021 in ClinicalTrials.gov (Zhang et al., 2019). For instance, PHY906 is based on Huang-Qin-Tang’s prescription for common gastrointestinal distress and has been studied for seven cancer types in clinical trials (Wang et al., 2011; Saif et al., 2014; Liu, 2015; Ganguly et al., 2019). ACT001 is an analog of parthenolide derivative from the shoots of feverfew (*Tanacetum parthenium*). It has been approved as orphan drug status by the FDA and is in phase I clinical trials for advanced glioblastoma in China (CTR20171274) and Australia (ACTRN12616000228482) (Zhang et al., 2012).

One of the main characteristics of TCM is that it considers the human body as a holistic system to achieve maximal synergistic effects and minimal side effects (Wang et al., 2012; Zhou et al., 2017; Ramsay et al., 2018). The holistic concepts proposed by the TCM theories thousands of years ago coincide with the system biology concepts in modern medicine (Bahari and Yavari, 2021). As an essential branch of system biology, network pharmacology approaches have attracted considerable attention because of their potential for understanding drug interactions in many complex diseases. Hence, system pharmacology modeling has also been widely applied in TCM to explore active ingredients or targets and to understand therapeutic mechanisms of action (Maetschke et al., 2014; Kibble et al., 2015), such as herb properties (Naghizadeh et al., 2020; Naghizadeh et al., 2021), herb combinations (Vanunu et al., 2010; Hsieh et al., 2011; Wang et al., 2021), TCM diagnosis, and symptoms (Ma et al., 2010; Xie et al., 2018). The construction of networks in TCM mainly consists of associations between five main entities, including formulae, herbs, ingredients, targets, and diseases. Based on the network’s topology, familiar patterns or important nodes can be detected by various algorithms in network analysis. Furthermore, biological pathways or gene ontology (GO) functional terms can be inferred to discover potential mechanisms of actions (MOAs) of active ingredients in TCM (Wang et al., 2021).

Thanks to the rapid development of molecular profiling technologies (Xu et al., 2021), increasing data at multiple omics levels for both herbs and ingredients were available (Guo et al., 2020). These data were curated, standardized, and stored as databases to benefit researchers with valuable resources (Xu et al., 2021). Multiple databases have been established recently, providing diverse information for TCM herbs or ingredients (Lagunin et al., 2014; Lee et al., 2019). For instance, recent reviews summarize the databases and tools currently used for TCM research (Zhang et al., 2019). However, fewer of them have compared the overlap of these databases. Furthermore, coverage of the trends of TCM databases to advance network pharmacology is limited. We first determined their overlapping herbs, ingredients, and herb-ingredient pairs based on all the available data downloaded from major TCM databases published since 2006. Secondly, we reported the developing trend of TCM databases from the perspective of network pharmacology, such as network construction and analysis, external linking databases, and absorption, distribution, metabolism, and excretion (ADME) properties. Finally, we proposed a few promising directions and approaches for improving and developing TCM databases.

## 2 Overview of the significant TCM databases

**FIGURE 1:**
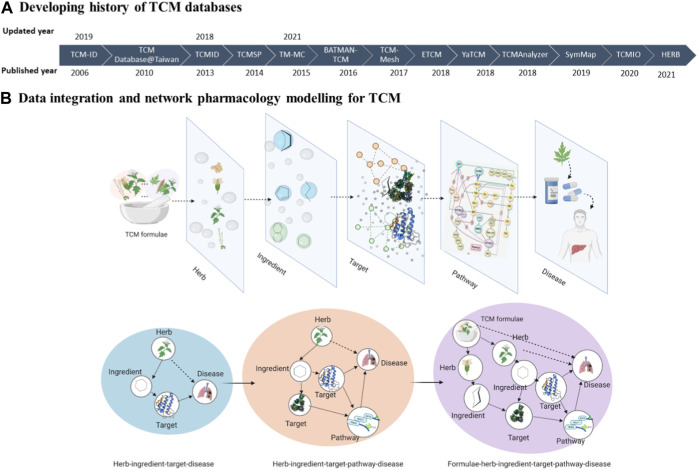
The schematic of this review. **(A)** Developing history of TCM databases. **(B)** Data integration and network pharmacology modelling for TCM.

Here, we briefly described 14 TCM databases developed during the last two decades. These databases are under active development and, therefore, are expected to capture the recent updates in the TCM research (Figure 1).

### 2.1 TCM-ID

TCM-ID (Chen et al., 2006) (http://bidd.group/TCMID/) was initialized in 2006, including prescriptions (*n* = 1,588), constituent herbs (*n* = 1,313), herbal ingredients (*n* = 5,669), and their corresponding molecular information (*n* = 3,725). The database currently consists of 7,443 prescriptions, 2,751 herbal medicines, and 7,375 chemical ingredients. In particular, the drug-target information for the ingredients has been obtained from an *in silico* method named INVDOCK (Chen and Zhi, 2001) and, more recently, from experimental validation of bioactivity assays.

### 2.2 Database@taiwan

Database@taiwan (Chen, 2011) (http://tcm.cmu.edu.tw/) was developed in 2011 and initially contained 20,000 pure compounds and 435 TCM herbs. The number of compounds has increased to about 61,000 more recently. Although virtual screening and molecular simulation approaches are commonly used for drug discovery, their applications are rare in TCM. Therefore, Database@taiwan aimed to support virtual screening or molecular simulation with the molecular structure of ingredients in TCM.

### 2.3 TCMSP

TCMSP (Ru et al., 2014) (https://old.tcmsp-e.com/index.php) was published in 2012 and then updated in 2014, including 499 herbs, 29,384 ingredients, 3,311 targets, and 837 associated diseases.

TCMSP aims to establish an efficient systems pharmacology platform to integrate various information, such as pharmacochemistry, ADME properties, drug-likeness, and drug targets. In the TCMSP database, a comprehensive network between herbs–compounds–targets–diseases (H–C–T–D) was created to help illustrate the MOAs of TCM herbs, understand the rationale of TCM theory, and discover herb-derived drugs. TCMSP is also one of the first TCM databases that systematically reported ADME properties to enable the filtering of the ingredients that have poor oral absorbability and low drug-likeness.

### 2.4 TCMID

TCMID (Xue et al., 2013) (http://www.megabionet.org/tcmid/) integrates the data from Database@Taiwan and other databases and the literature. TCMID was updated in 2018, including 49,000 prescriptions, 8,159 herbs, 25,210 ingredients, 3,791 diseases, 6,828 drugs, and 17,521 targets. TCMID visualizes interactions between formulae, herbs, components, and their target proteins to support the network modeling.

### 2.5 BATMAN-TCM

BATMAN-TCM (Liu et al., 2016) (http://bionet.ncpsb.org.cn/batman-tcm/) is a bioinformatics tool for analyzing molecular mechanisms of TCM published in 2015.

BATMAN-TCM focuses on understanding the multi-component, multi-target, and multi-pathway combinational therapeutic mechanism of TCM. To explore the molecular mechanism of combinations of formulae or herbs, BATMAN-TCM provides the predicted targets for TCM ingredients. Also, BATMAN-TCM is a bioinformatics tool that performs functional analyses and visualization of targets, such as biological pathways, GO functional terms, and disease enrichment analyses.

### 2.6 TM-MC

TM-MC (Kim et al., 2015a) (http://informatics.kiom.re.kr/compound/) extracted 14,000 chemical compounds from 536 medicinal materials and 4,000 journal articles in MEDLINE and PubMed Central (PMC). Although many TCM databases provide diverse information, the sources of such information are seldom reported; thus, it is difficult to verify them. To solve this limitation, TM-MC aimed to construct a database to provide detailed sources of information in PubMed, PubChem, and ChemSpider for each herb-ingredient pair.

### 2.7 TCM-Mesh

TCM-Mesh (Zhang et al., 2017) (http://mesh.tcm.microbioinformatics.org) was published in 2017, including 6,235 herbs, 383,840 compounds, 14,298 genes, 6,204 diseases, 144,723 gene-disease associations and 3,440,231 pairs of gene interactions. TCM-Mesh was designed to integrate various resources and is intended to serve as a more comprehensive and user-friendly platform for network pharmacology analysis. In addition, TCM-Mesh provides the toxicity and side effects of ingredients, which is vital for safety assessments during the application of TCM. In total, 163,221 side effect records (1,430 ingredients and 6,123 side effects) were extracted from TOXNET (Fowler and Schnall, 2014) and SIDER (Kuhn et al., 2016).

### 2.8 TCMAnalyzer

TCMAnalyzer (Liu et al., 2018) (http://www.rcdd.org.cn/tcmanalyzer) was developed in 2017, covering 1,493 formulae, 618 TCM medicine, and 16,437 ingredients.

Many ingredients and their interactions with biological receptors are unknown, which makes it difficult to determine the molecular mechanisms of action. To solve this problem, TCMAnalyzer intended to identify the active ingredients, protein targets, therapeutic mechanisms, and critical structural fragments responsible for the therapeutic activities by cheminformatics and bioinformatics approaches. Compared with other TCM databases, TCMAnalyzer deepens the understanding of the structure of TCM ingredients by substructure-searching tools, similarity-searching tools, and scaffold-searching tools.

### 2.9 YaTCM

YaTCM (Li et al., 2018) (http://cadd.pharmacy.nankai.edu.cn/yatcm/home) was published in 2018 and contained 47,696 natural compounds, 6,220 herbs, 18,697 targets (including 3,461 therapeutic targets), 1,907 predicted targets, 390 pathways, and 1,813 prescriptions. Compared with other TCM databases, YaTCM supports unique analytical tools, including similarity and substructure searching for potential structures and identifying similar biological functions between herb pairs.

### 2.10 ETCM

ETCM (Xu et al., 2019) (The Encyclopedia of Traditional Chinese Medicine) ( http://www.tcmip.cn/ETCM/) is a web server tool established in 2018 for the network analysis of TCM, including herbs (*n* = 402), formulae (*n* = 3,959) and ingredients (*n* = 7,284). ETCM has some unique characteristics. For instance, the annotation information for herbs and formulae is richer than other databases as ETCM includes not only the habitat and quality control information of herbs but also various drug-likeness information of the ingredients. ETCM also has improved functions for network analysis and visualization.

### 2.11 SymMap

Clinical symptoms in TCM are vital for diagnosis and treatment. To study the TCM symptoms more systematically, SymMap (Wu et al., 2019) (https://www.symmap.org/) was established in 2019 as an integrative database that maps symptoms in TCM to modern symptoms and diseases, covering 1,717 TCM symptoms, 499 herbs, 961 modern symptoms, 5,235 modern diseases, 4,302 targets, and 19,595 ingredients.

### 2.12 HERB

The HERB (Fang et al., 2021) database (high-throughput experiment and reference-guided database of traditional Chinese medicine) (http://herb.ac.cn/) is one of the few databases that contain transcriptomic profiles for herbs and ingredients. Established in 2020, HERB has 6,164 gene expression profiles of TCM herbs or ingredients from 1,037 high-throughput experiments. In addition, 12,933 targets and 28,212 diseases were further linked to 7,263 herbs and 49,258 ingredients by statistical inference. Moreover, the gene targets (*n* = 1,241) and modern disease indications (*n* = 494) for 473 herbs/ingredients were manually collected from 1,966 scientific references.

HERB aimed to help researchers build a high-quality pharmacology network by gene expression data, thus uncovering evidence-based associations between TCM and modern drugs. In addition, HERB also manually collects high-confidence compound-target interactions and herb-disease associations from the literature.

### 2.13 TCMIO

Numerous herbs or ingredients have been reported to have immunomodulatory functions and antitumor effects by targeting the immune system. However, their underlying mechanisms remain unclear. To tackle this issue, TCMIO (Liu et al., 2020) (Traditional Chinese Medicine on Immuno-Oncology, http://tcmio.xielab.net) was recently developed in 2020, including 1,493 prescriptions, 618 TCM medicine, 16,437 ingredients, and 32,847 TCM-ingredient-associations.

TCMIO was designed to explore the role of TCM in modulating the cancer immune microenvironment. Unlike other databases, TCMIO focuses only on formulae, herbs, ingredients, targets, and diseases related to immuno-oncology.

### 2.14 TCMSID

Traditional Chinese Medicine Simplified Integrated Database (TCMSID, https://tcm.scbdd.com/home/index/) covers 499 herbs in the Chinese pharmacopeia and 20,015 ingredients. TCMSID evaluates the structural reliability of all ingredients and their possibility of exerting pharmacological effects. In addition, the potential targets of ingredients are predicted by multiple target prediction tools.

## 3 Systematic comparison of TCM databases

### 3.1 Sizes of TCM entities

**FIGURE 2:**
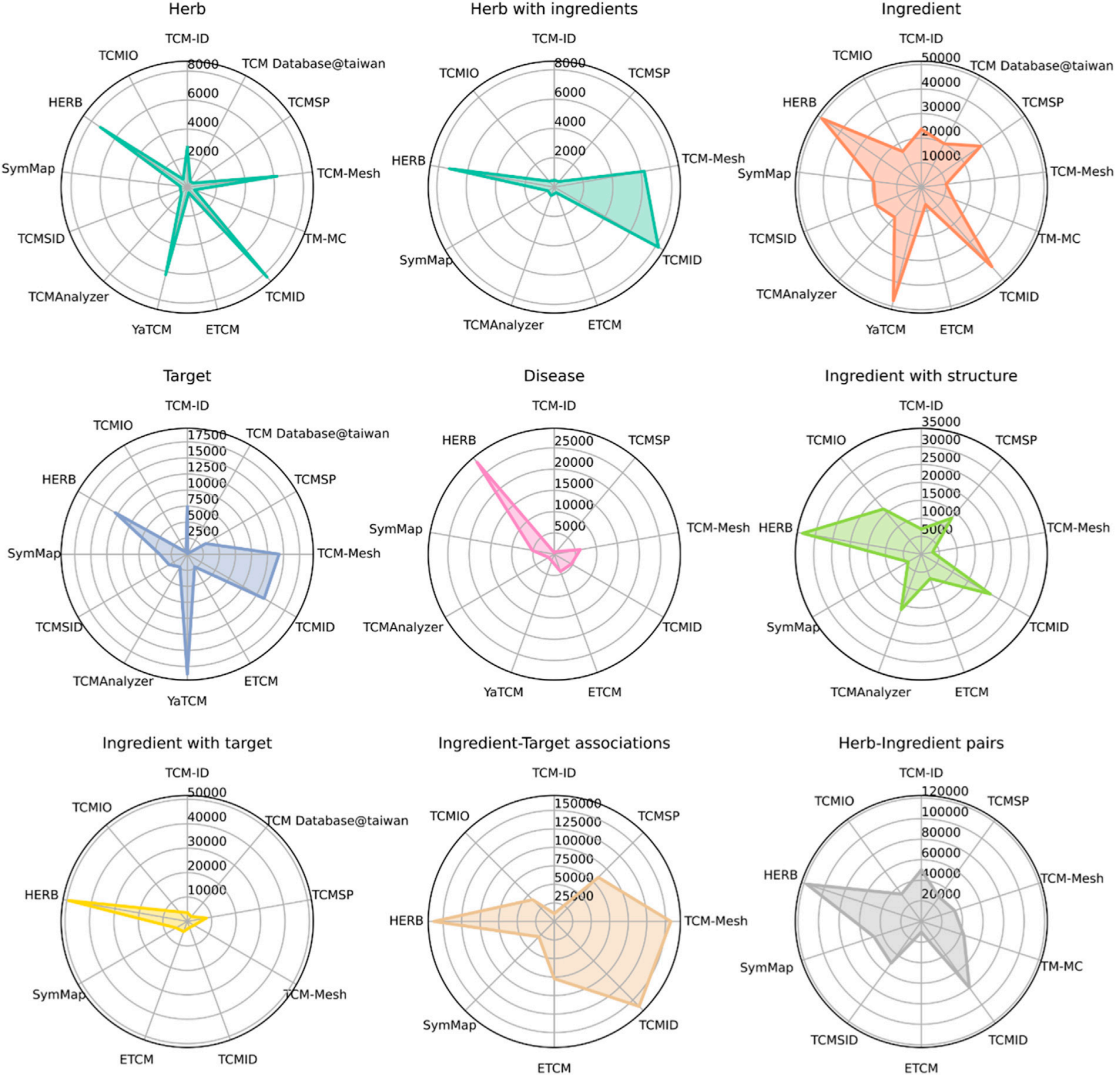
Summary of data sizes for multiple TCM entities, including herbs, herbs with ingredient information, ingredients, targets, diseases, ingredients with structure information, ingredients with target information, ingredient-target interactions, and herb-ingredient interactions. Note that a database does not necessarily contain all these entities’ information. Only the databases with the corresponding data entities are shown for each plot.

We compared the number of data points in the TCM databases for nine entities, including herbs, herbs with at least one ingredient, ingredients with structure information, ingredients with at least one target, herb-ingredient pairs, ingredient-target pairs, targets, and diseases. As shown in Figure 2, HERB has the most extensive coverage in eight of these nine entities, except for the number of targets, with 7,263 herbs, 49,258 ingredients, 12,933 targets, and 28,212 diseases. As one of the newly developed databases, HERB integrates information from the other databases, leading to a much more extensive collection of targets and diseases. Other databases, including TCMID, TCM-Mesh, and YaTCM, have a similar number of herbs (*n* > 6,000) as compared to the remaining databases (*n* < 2,000). Similarly, the top TCM databases with the largest ingredients are HERB, YaTCM, TCMID, and TCMSP (*n* > 30,000), while TCM-Mesh has fewer ingredients. In addition, HERB and TCMID have the most abundant herb-ingredient pairs (*n* > 8,000).

In brief, TCM databases have experienced a fast development in recent decades, accumulating information for ∼8,000 herbs, ∼50,000 ingredients, and ∼120,000 herb-ingredients pairs. Moreover, ∼150,000 ingredient-target associations were predicted by computational methods.

### 3.2 Shared herbs and ingredients

**FIGURE 3:**
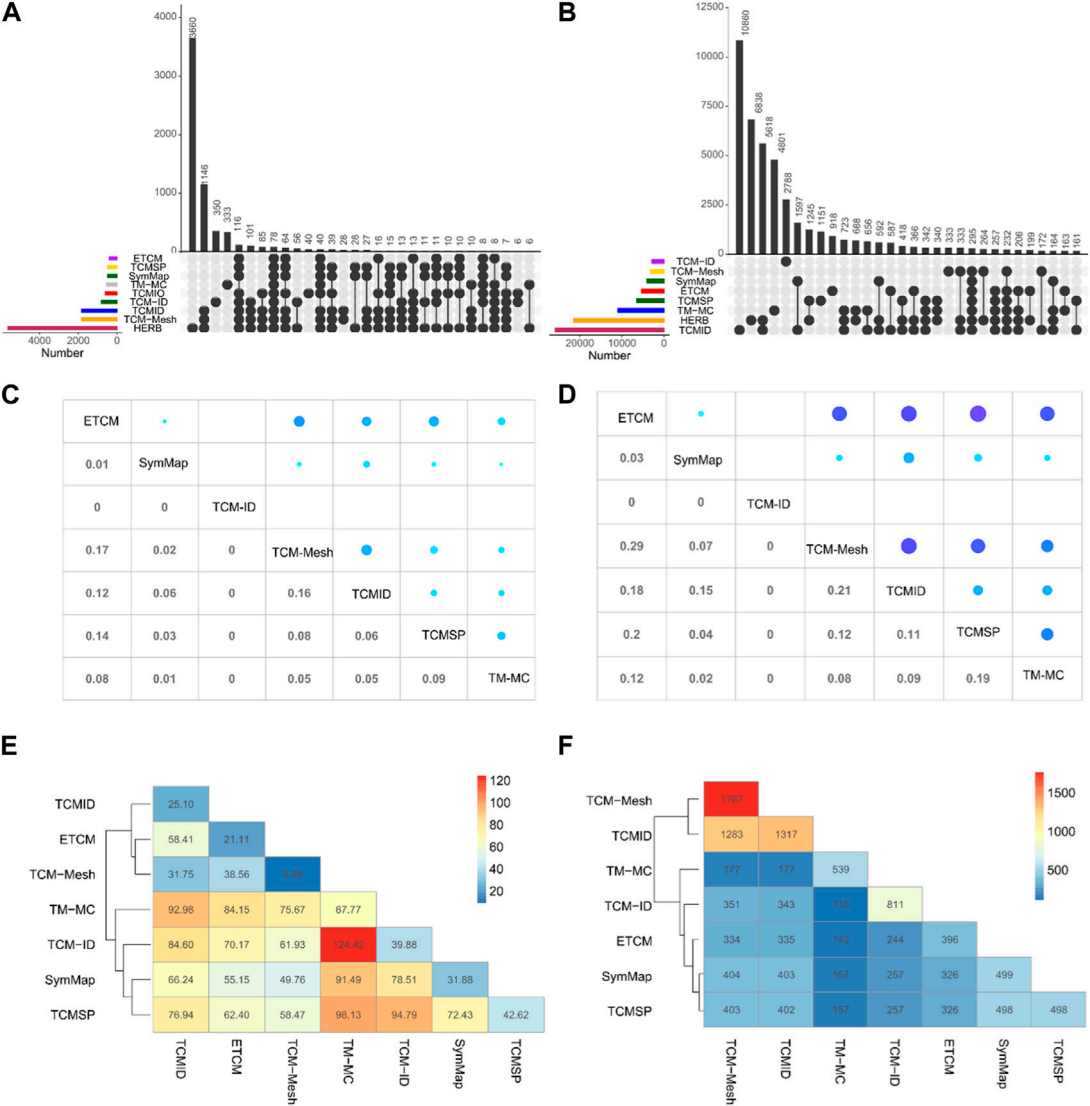
Overlapping of herbs and ingredients between TCM databases. Upset plot for the shared herbs **(A)** and ingredients **(B)** among the TCM databases. The color bars at the bottom left represent the number of herbs or ingredients in each TCM database, which can be further collapsed into subclasses depending on whether a herb or an ingredient exists in one or more TCM databases. The vertical bars show the number of shared herbs or ingredients for a particular subset of TCM databases, as indicated by the connected lines below the x-axis between the databases. Average Jaccard coefficients **(C)** and overlap rates **(D)** of herb-ingredient relationships between the common herbs in seven TCM databases. The average value of shared herb-ingredient relationships **(E)** and number of pairwise common herbs **(F)** between seven TCM databases.

We determine the number of common herbs and ingredients to explore the overlap among the TCM databases. We matched herbs and ingredients by their Chinese names and PubChem IDs respectively, on the TCM databases for which the data can be downloaded. As shown in Figure 3A, HERB has the most unique herbs (*n* = 3,660), followed by TCM-ID (*n* = 350) and TM-MC (*n* = 333). There are only 78 herbs shared among nine databases, suggesting a minimal overlap. When excluding TM-MC, the overlap increases to 116 herbs. Furthermore, TCMID, TCM-Mesh, and HERB share more common herbs than the other databases (*n* = 1,146).

Compared with the overlap situation in herbs, the number of overlapped ingredients between eight databases is lower, with only 295 common elements (Figure 3B). In contrast, TCM databases contain a more significant number of unique ingredients (TCMID = 10,860, HERB = 6,838, TM-MC = 4,801, TCM-ID = 2,788, TCMSP = 1,151, and, ETCM = 918). TCMID and HERB shared the most common ingredients (*n* = 5,618). Generally, the consistency of the herb information contained among TCM databases is higher than that for ingredients.

### 3.3 Shared herb-ingredient pairs

Herbal ingredients are vital for exploring the TCM mechanisms at the molecular level. Therefore, we compare the herb-ingredient pairs between the TCM databases. We consider the average overlap rate and Jaccard coefficient across all the common herbs between a given pair of databases. Namely, for a common herb, A and B represent the set of ingredients of this herb in the two databases, respectively. The overlap rate is defined as (∥A∩B∥)/∥A∥ , where ∥A∩B∥ is the number of common ingredients and is further divided by all the number of ingredients of this herb in database A. Similarly, Jaccard index is defined as (∥A∩B∥)/(∥A∪B∥) .

As illustrated in Figures 3C, D, TCM-Mesh and TCMID have the maximum average Jaccard index (0.16), while TCM-Mesh and ETCM have the top average overlapped rate (0.29). ETCM has a relatively higher overlap rate with other databases, such as TCM-Mesh (0.29), TCMSP (0.20), TCMID (0.18), and TM-MC (0.12). In contrast, TCM-ID has no overlap with any of the other databases. We found that TM-MC tends to have more common herb-ingredient pairs with other TCM databases, with an average of 124.42 (Figure 3E). For example, for the 177 common herbs in TCM-ID and TM-MC, on average, 124 common herb-ingredient pairs can be identified. Furthermore, TCM-Mesh and TCMID share only 31.75 common herb-ingredient pairs, despite having 1,283 common herbs (Figure 3F). The distribution of shared herb-ingredient associations and the Jaccard index for ingredients of common herbs between TCM databases can be seen in Supplementary Figures S1, S2.

Taken together, we found a relatively low overlap of herbs and their ingredients between different databases, suggesting that a more unified knowledge base is needed to integrate these databases for further study.

### 3.4 Types of annotations

**FIGURE 4:**
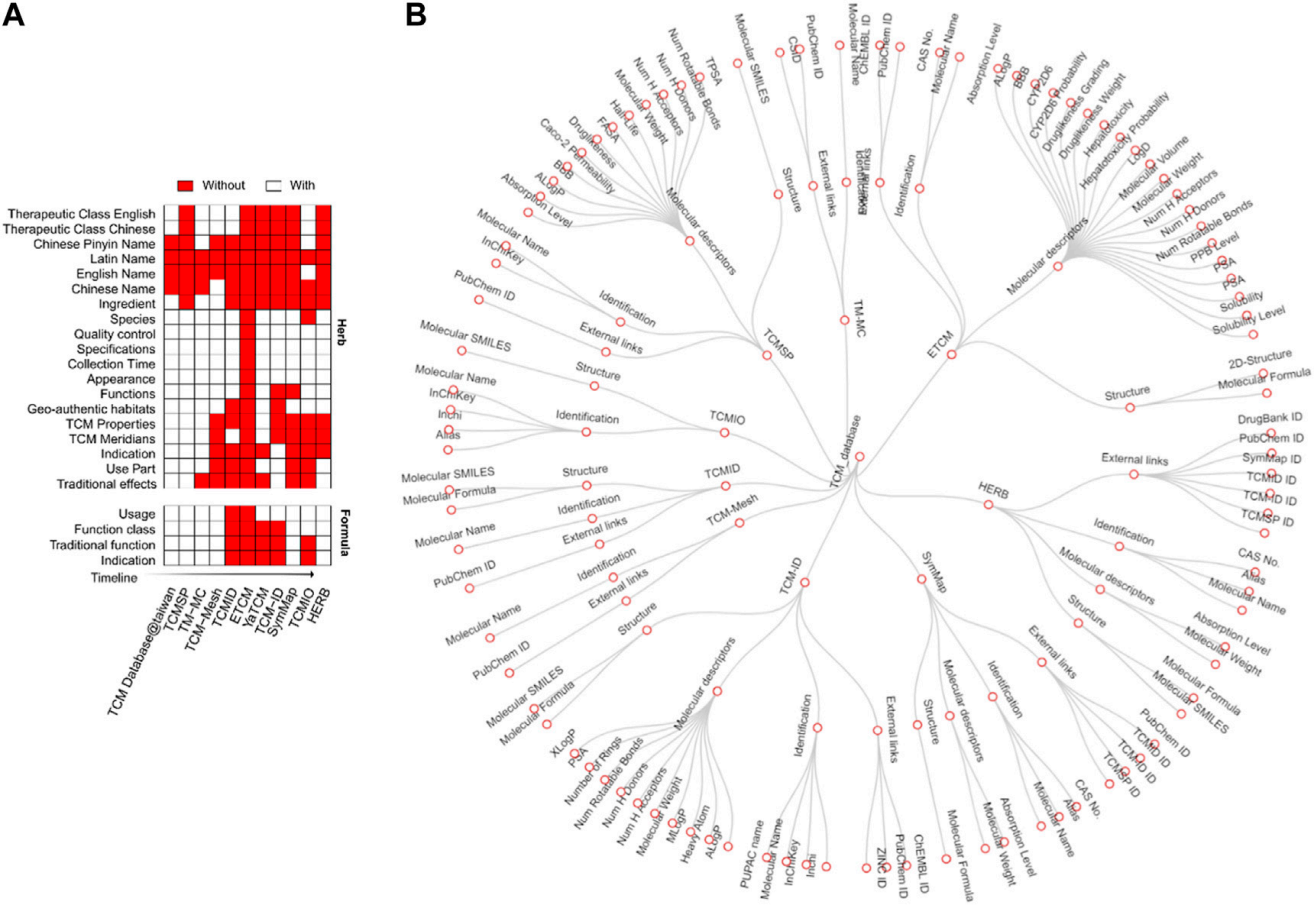
Types of annotations in different databases. **(A)** Annotation types for herbs (left) and formulae (right). In the heat map, rows are TCM databases, and columns are annotation items, shown in red when available. The databases were ordered by their publishing years from top to bottom. **(B)** Annotation tree for TCM ingredients. The nodes from inside to outside represent TCM databases, types of ingredient annotation, and their properties, respectively.

Annotation of TCM usually contains information about formulae, herbs, ingredients, targets, and disease indications. With the development of TCM databases, annotation types have become increasingly available for many herbs. For example, TCM database@taiwan, one of the earliest TCM databases, only contained the names of TCM herbs. After that, TCMSP, published in 2014, provided therapeutic classes of herbs and their ingredients to support more sophisticated network pharmacology analyses. More recently, TCM databases contain more annotations, such as TCM properties, meridians, disease indications, and therapeutic effects (Figure 4A).

Another improvement is the annotation of the TCM formula, a unique concept that specifies how herbs can be combined to treat diseases. TCMID was the first database containing TCM formula information, including usage, classification, and indication. The therapeutic effects of one formula can be classified by the Western medicine system as “indication” and by the traditional medicine system as “function classes” according to their specific “traditional function.” For example, herbs with functional effects nourish the temper and replenish the heart, which belongs to the function class tonic medicine. So far, five databases are providing formulas, including TCM-ID, TCMID, YaTCM, ETCM, and TCMIO (Figure 4A). Although the complete species names are vital to avoid ambiguity in the use of herbal medicine, only the ETCM and ICMIO databases provide species classification. On the other hand, TCM-ID can link the prescription component by its Barcode ID into the Barcode of Life Data Systems (BOLD) database (Ratnasingham and Hebert, 2007). However, the DNA barcoding data was typically determined for two or three genes, which are limited in differentiating plants in the same genus. To improve the quality of the TCM databases, it is necessary to apply standardized reference resources such as Medicinal Plant Names Services (http://mpns.kew.org/mpns-portal/) or Plants of the World Online (http://www.plantsoftheworldonline.org) to reduce the ambiguity about the identities and names of the species. Furthermore, as an important quality control step, DNA sequencing of a comprehensive panel of marker genes should be provided to avoid species misidentification (Rivera et al., 2014).

An annotation tree was plotted to better illustrate the annotation of ingredients in different databases (Figure 4B). There are four main annotation types: ADME properties, external links, structure, and names. For each annotation type, there exists a different number of items. For example, SMILES, PubChem ID, and Mol2 are commonly used to represent the structure of ingredients. Physiological features such as molecular weights and solubility are generally reported for ADME properties. ADME gains increasing interest in the research of TCM as TCM is administered by decoction, which triggers complex absorption, distribution, and metabolism processes. It is known that TCM ingredients can mimic the metabolites of the human body to treat diseases (Kim et al., 2015). Currently, three databases provide ADME properties (Figure 4B), including TCMSP, YaTCM, and ETCM. For example, TCMSP provides 12 ADME properties systematically, such as oral absorbability, half-life, drug-likeness, Caco-2 permeability, blood-brain barrier, and Lipinski’s rule of five. These properties are considered to be essential for drug discovery in TCM. YaTCM focuses on 50 fundamental ADME properties, including four physicochemical descriptors and 48 ADME descriptors. ETCM reports around ten physical-chemical properties and six ADME properties, including blood-brain barrier penetration, CYP450 2D6 inhibition, hepatotoxicity, human intestinal absorption, plasma protein binding, and the quantitative estimate of drug-likeness (QED). Considering these ADME properties of ingredients in the study of network pharmacology could help to prioritize the potential compounds for drug discovery.

In summary, although the annotation for herbs and ingredients has also been improved, the ADMET properties were only found in four databases, with notable differences.

### 3.5 Network pharmacology modeling to explore the mechanisms of action

Protein targets of ingredients are essential for the MOAs of disease treatment in TCM (Chen et al., 2003). In TCM databases, the validated ingredient-target interactions are mainly extracted from four resources, including 1) Text mining from the literature, including TCM-ID and HERB; 2) the ChEMBL database (Gaulton et al., 2017), including TCM-ID, TCMAnalyzer, and TCMIO; 3) the STITCH database (Kuhn et al., 2008), including TCMID and TCM-Mesh; and 4) the HIT database (Ye et al., 2011), including TCMSP.

In addition to validated targets, most TCM databases provide predicted targets from computational methods (Table 1). In databases published before 2014, docking methods are commonly used. For example, TCM-ID implemented a ligand-protein inverse docking strategy called INVDOCK to search targets in the Protein Data Bank (PDB) (Chen and Zhi, 2001). Database@taiwan also predicts compound-target interactions by virtual screening with docking and molecular dynamics simulations. However, docking-based virtual screening approaches are usually demanding on proteins’ computational resources and 3D structures. Therefore, more TCM databases began to implement similarity-based target prediction models. For instance, TCMSP utilizes a SysDT model (Yu et al., 2012), and YaTCM utilizes a multi-voting chemical similarity ensemble approach (Wang et al., 2016). TCMIO relies on a balanced substructure-drug-target network-based inference [bSDTNBI (Wu et al., 2016)] approach based on heat diffusion modeling. In TCMSID, the potential targets of ingredients are predicted by metaTARFISHER (https://metatarget.scbdd.com/), a tool that provides multiple algorithms, including SwissTargetPrediction (Gfeller et al., 2014; Daina et al., 2019), SEA (Wang et al., 2016), HitPickV2 (Hamad et al., 2019), Polypharmacology Browser and Polypharmacology Browser 2 (Awale and Reymond, 2019). In contrast, HERB applies Fisher’s exact test to infer the targets directly from the manually collected 1,966 references rather than docking or similarity-based target prediction.

**TABLE 1:** Network pharmacology modeling in TCM databases.

Database	Last update year	Open access	Source of targets	Number of herbs- or ingredient-target pairs	Network modeling entities	Functional analysis
TCM Database@taiwan	2010	Yes	Virtual screening by docking and molecular dynamics	/	/	/
TCMSP	2014	Yes	HIT SysDT	84,260 ingredient-target pairs (7,947 ingredients, 1,079 targets)	Herbs, ingredients, targets, and diseases	/
BATMAN-TCM	2016	Yes	Similarity-based target prediction	/	Formula, herbs, ingredients, targets, pathways, and diseases	KEGG biological pathways
						Gene Ontology (GO) functional terms
TCM-Mesh	2017	No	STITCH	/	Prescriptions, herbs, ingredients, targets, and diseases	/
TM-MC	2015	Yes	/	/	/	/
TCMID	2018	No	STITCH	/	Prescriptions, herbs, ingredients, targets, and diseases	/
ETCM	2018	Yes	MedChem Studio (chemical fingerprint similarity)	/	Prescriptions, herbs, ingredients, targets, and diseases	KEGG biological pathways
						Gene Ontology (GO) functional terms
YaTCM	2018	No	Multi-voting chemical similarity ensemble approach	/	Prescriptions, herbs, ingredients, targets, and diseases	KEGG biological pathways
						Gene Ontology (GO) functional terms
TCMAnalyzer	2018	No	ChEMBL	/	Ingredients, targets, and diseases	KEGG biological pathways
						Gene Ontology (GO) functional terms
TCMSID	2022		metaTARFISHER	/	/	/
SymMap	2019	Yes	/	/	TCM symptoms, modern medicine symptoms, herbs, ingredients, targets, and diseases	/
TCM-ID	2019	Yes	Text mining	78,117 herb-human target pairs (463 targets, 1,323 herbs)	/	Gene Ontology (GO) functional terms
			ChEMBL	23,946 herb-microbe protein pairs (305 targets, 1,209 herbs)		
			Ligand–protein inverse docking	10,750 validated ingredient-target pairs from experiments (1,656 ingredients, 667 targets)		
HERB	2021	Yes	Text mining	291 herb-target pairs (39 herbs and 182 targets)	Herbs, ingredients, genes, and diseases	Differentially expressed qaaqgenes
			Fisher’s exact test	4,815 ingredient-target pairs (370 ingredients and 1,205 targets)		
TCMIO	2020	Yes	ChEMBL	/	Prescriptions, herbs, ingredients, and targets	Gene Ontology (GO) functional terms
			Balanced substructure-drug-target network-based inference (bSDTNBI)			KEGG biological pathways

Many TCM databases harbor a mixture of experimentally validated and computationally predicted targets. In addition, the targets for herbs and formulae are usually considered as a union of targets from their ingredients, which is not necessarily true as their underlying target interactions are much more complex. Specific target prediction models at the TCM herb or formula levels are still in the early stages, with a few examples (Gu and Lai, 2020).

### 3.6 Disease-related properties

To help understand the rationale of TCM, most databases classify herbs and their disease indications inferred from the putative targets. Furthermore, the disease indications are annotated with commonly accepted standard terms. For example, the TCM-ID database has 153 functional classes, 380 disease indications, and 366 ICD-11 categories. In detail, there are 114,651 formulae-indication pairs involving 7,440 formulae and 380 indications. There are also 17,624 functions, covering 6,465 formulae and 4,629 functions. Similarly, in TCMSP, the disease information (2,387 target-disease pairs) was established by retrieving 2,387 targets and 84,260 compound-target pairs from the TTD database (Chen et al., 2002) (https://doi.org/10.1093/nar/gkp1014) and PharmGKB (Barbarino et al., 2018) (https://www.pharmgkb.org/). In contrast, the gene-disease associations in TCM-Mesh were collected from the GAD database (Becker et al., 2004). The ETCM database utilizes multiple resources, such as Phenotype Ontology (Köhler et al., 2017), Online Mendelian Inheritance in Man (OMIM) (Amberger and Hamosh, 2017), Database of gene-disease associations (DisGeNET) (Piñero et al., 2015) and ORPHANET database (Pavan et al., 2017). In YaTCM, the disease indication of formulae and herbs is based on the therapeutic phenotypes rather than their target genes. Unlike the previously mentioned databases that rely on targets for disease classification, the SymMap database aims to map TCM symptoms into disease indications directly (Xie et al., 2020). Namely, SymMap first curates 1,717 TCM symptoms of 499 herbs and then maps them to 961 symptoms in modern medicine. These current symptoms were finally linked to 5,235 diseases. As multiple levels of associations for formula, herbs, ingredients, targets, and diseases have been established, network pharmacology modeling has become a standard technique to tackle the mechanisms of action of TCM, where the KEGG pathway and GO analyses have been commonly used.

In summary, despite multiple databases that have provided ingredient-disease, herb-disease, and formulae-disease associations, many of them were inferred from computational approaches. In contrast, the disease symptom classifications are well-defined, although they differ from those used in mainstream medicine. As a result, phenotypic-based drug discovery (PDD) is a favorable strategy for finding new indications for TCM.

### 3.7 Interconnections of TCM databases

**FIGURE 5:**
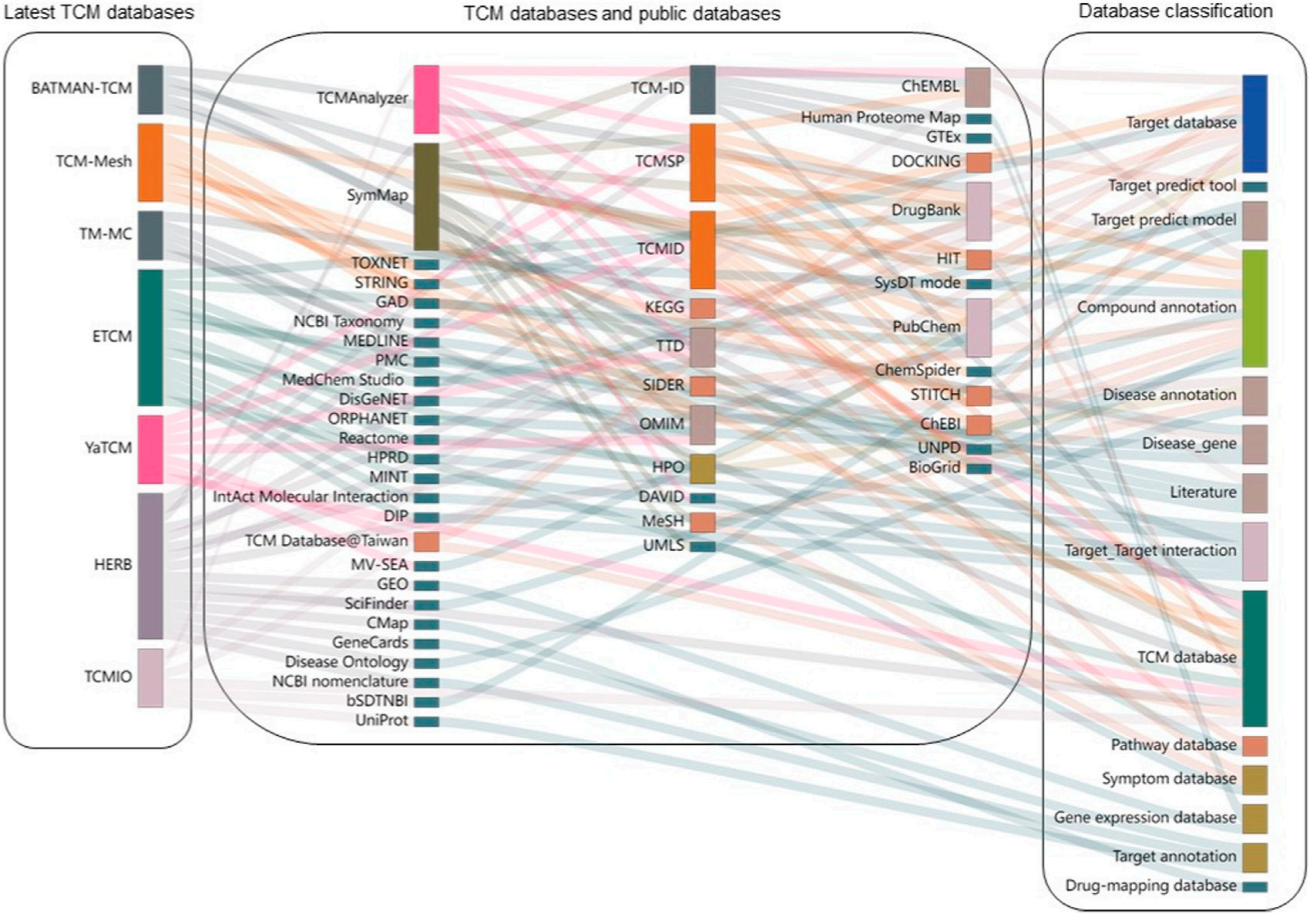
Interconnections of TCM databases and their data sources. Each TCM database is shown as bars on the left side, connecting to their data sources in the middle panel. These data sources are further grouped into different categories on the right side. The height of each rectangle represents the frequency with which it was linked to other databases.

The relationships between these TCM databases are shown in Figure 5. TCMSP, TCM-ID, and TCMID were published before 2014 and were further utilized by other more recent databases, such as TCMAnalyzer and SymMap. HERB integrated information from the most significant number of other TCM databases, followed by ETCM and SymMap. We found that the TCM databases utilize multiple data sources that are grouped into four categories, including:

1) Target databases and tools (e.g., target prediction tools, target-target interaction, and annotation databases)
2) Compound databases and tools (e.g., compound annotation)
3) Disease databases and tools (e.g., disease annotation, disease genes, pathways, and symptom databases)
4) Others (e.g., scientific literature databases and gene expression databases)

Many data sources are commonly utilized in multiple TCM databases, such as PubChem, DrugBank, and ChEMBL, to annotate compounds and targets. As shown in Figure 5, the most extensively involved data source is compound annotation databases (*n* = 10), including PubChem and DrugBank. In addition, various target-related databases (*n* = 9), such as DrugBank, OMIM, and ChEMBL, are also utilized. However, there are quite a few data sources that are used by specific TCM databases. For example, Reactome (Matthews et al., 2009), HPRD (Peri et al., 2003), MINT (Zanzoni et al., 2002), DisGeNet, and GAD are only used for ETCM, while GEO (Barrett et al., 2013), CMAP (Lamb et al., 2006), and GeneCards (Safran et al., 2010) are unique resources for HERB. Therefore, it is expected that connecting TCM databases to other public medicinal databases via compound-target and target-disease associations can enhance our understanding of herbal medicine at the molecular level.

## 4 Discussions

The lack of information has been a limiting factor for exploring and applying TCM. With the development of computational tools, increasingly comprehensive TCM databases have been developed. To fully use all the available databases, it is essential to compare them comprehensively. Although there are several comparative studies, most of them covered TCM databases published before 2018, and little comparison about ingredients and herb-ingredient pairs has been made.

In this study, we comprehensively analyze 14 major TCM databases. We compared the recent trends of TCM data curation, including their primary functions, annotations, network analysis, and visualization tools. We searched for the herbs by their Chinese names and found that the information about their ingredients differ across different databases. We also found that these TCM databases provide ununified annotation for herbs or ingredients, especially for the structure information of the ingredients, making it challenging to integrate data from them. Furthermore, we summarized novel multi-omics and advanced bioinformatics approaches that have been applied in the study of TCM, such as symptoms or gene expressions, which may provide new insights for drug discovery from TCM. We foresee that such a comparative study would help improve the understanding of data complexity that may ultimately motivate more efficient and more standardized strategies towards the digitalization of TCM.

TCM databases have been developed rapidly. Initially, the databases contained only basic information (e.g., TCM-ID, TCMdatabse@taiwan, and TCMSP), and increasing volumes of data have been added to enable a network pharmacology visualization (e.g., TCMID and TCM-Mesh), and functional analyses (e.g., ETCM and YaTCM). A notable trend is that more specific databases, such as SymMap, have been intended for symptoms and HERB mainly for transcriptional data. In addition, the ingredient search functions are becoming more flexible and powerful. With these tools, ingredients can be searched in TCM databases through direct keywords such as herbs, SMILES, or names and structures or substructures. If two compounds are similar in structure, they usually have identical properties or biological activities (Jafari et al., 2020). Hence, a comparison of the structural similarity between TCM ingredients and known drugs is needed. Several TCM databases have provided such a functionality. For instance, YaTCM uses the likeness of KEGG (Kanehisa et al., 2017) pathways to search potential ingredients, while TCMAnalyzer is based on molecular fingerprints’ similarity. Furthermore, drug-target prediction methods are commonly used in BATMAN-TCM and TCMID.

Harmonization of terminology is critical for improving the quality of TCM databases. Among these databases, BATMAN-TCM, TM-MC, HERB, and TCMIO provide scientific binomials for plants. Particularly, TCMIO provides scientific plant names, coupled with the names of publishing authors, to avoid potential ambiguity. As shown in Figure 4, Latin names of the herbs were commonly found across the databases. However, the majority of them were adopted from Pharmacopoeia to refer to herbal substances. These pharmacopeia names were not as precise as scientific botanical nomenclature. To ensure a better standardization of herbal substances, we recommend the use of the Medicinal Plant Names Services (http://mpns.kew.org/mpns-portal/) for nomenclatural indexing and references. On the other hand, the information on used parts was found in five databases, including TCM-Mesh, TCMID, ETCM, SymMap, and TCMIO, while the location and time of herb harvesting is available only in ETCM. Furthermore, we found that these TCM databases commonly lack information on the fingerprinting protocols, such as high-performance liquid chromatography (HPLC), gas chromatography (GC), and mass spectrometry (MS). According to the Consensus statement on the Phytochemical Characterization of Medicinal Plant extract (ConPhyMP) (Heinrich et al., 2022) (https://ga-online.org/best-practice), fingerprinting protocols contain essential information to ensure the reproducibility and interpretation of herb extract characterization. The current lack of such information across the TCM databases presents a critical limitation to reusing the data for more integrative analyses. Therefore, to improve the sharing of data and resources for the TCM research community, the FAIR (Findable, Accessible, Interoperable, and Reusable) principle should be carefully followed, similar to the data curation efforts for modern medicine (Almada et al., 2020; Tanoli et al., 2022).

Recently, many studies have performed high-throughput transcriptomic profiling for ingredients, herbs, and formulas. HERB is one of the first TCM databases to provide high-throughput gene expression data for herbs and ingredients, mainly from the GEO database. The differentially expressed genes (DEGs) were obtained by comparing samples treated with ingredients or herbs and control samples. These DEGs will lead to identifying pathways that are affected by TCM. Compared with the putative targets, the pathways derived from gene expression data may be more reliable to represent the holistic effects of specific herbs or ingredients. Therefore, we foresee that the increasing availability of molecular profiling data may open opportunities for more advanced bioinformatics and machine learning approaches to tackle the complexity of TCM.

In conclusion, our study covered an extensive collection of commonly used TCM databases. Also, the developing trends in TCM databases were summarized in the aspects of their primary functions, annotations, and network analysis. More importantly, we compared their overlaps of herbs, ingredients, and herb-ingredient associations. We found that TCM databases provide different complementary sets of information, suggesting the necessity of TCM database harmonization. Our comparison of TCM databases would help to deepen the understanding of TCM databases and to integrate a diversity of data efficiently from TCM databases.

## Data Availability

The original contributions presented in the study are included in the article/Supplementary Material, further inquiries can be directed to the corresponding authors.

## Supplementary material

SUPPLEMENTARY FIGURE S1 The distribution of shared herb-ingredient associations of common herbs between TCM databases.

SUPPLEMENTARY FIGURE S2 The distribution of the Jaccard index for ingredients in common herbs between TCM databases.
