# Supplementary figures and images for: A critical assessment of Traditional Chinese Medicine databases as a source for drug discovery
Source: Front Pharmacol. 2024 Apr 26;15:1303693. doi: 10.3389/fphar.2024.1303693 (PMC11082401; doi:10.3389/fphar.2024.1303693)

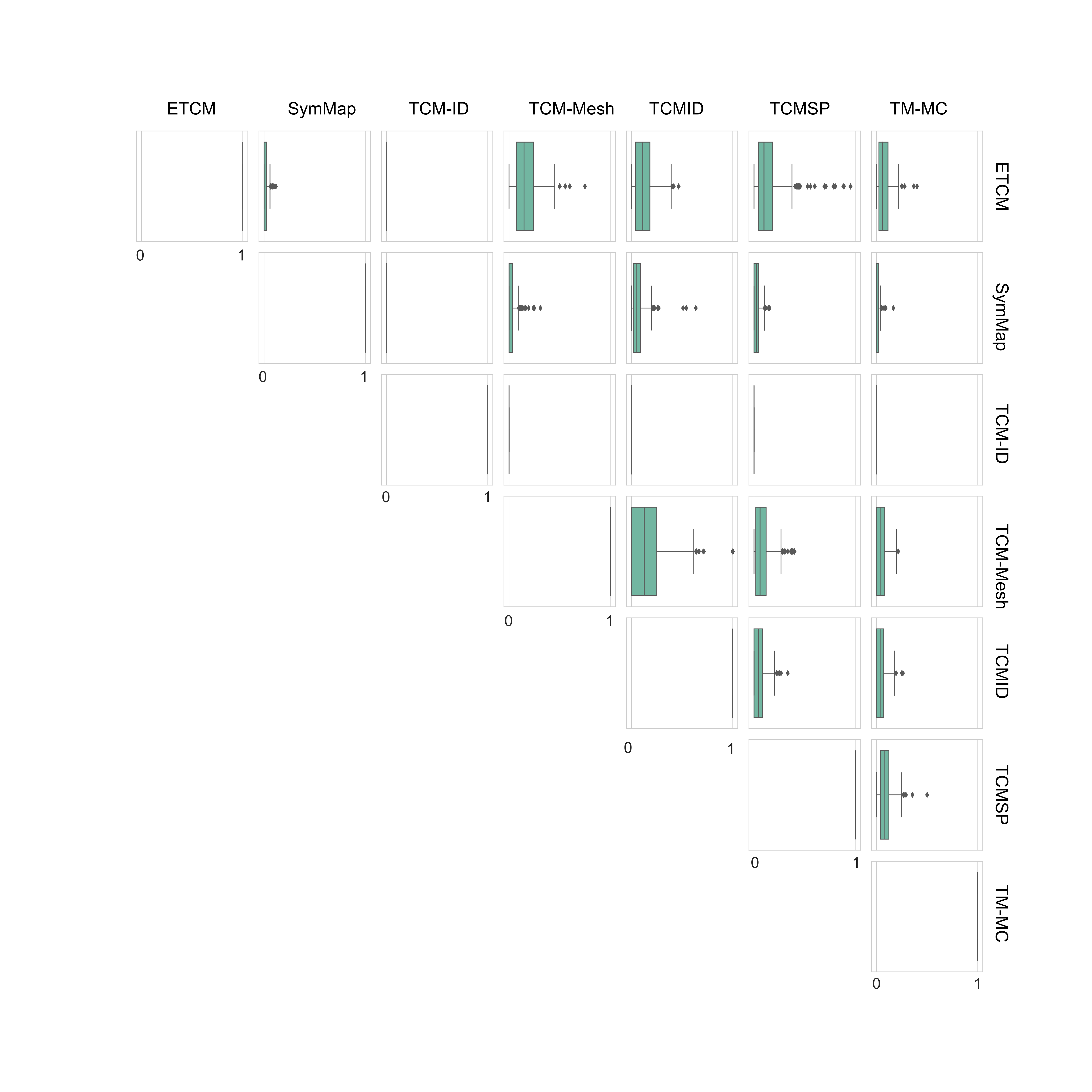

Supplement: Supplementary file 1 [file Image2.png]

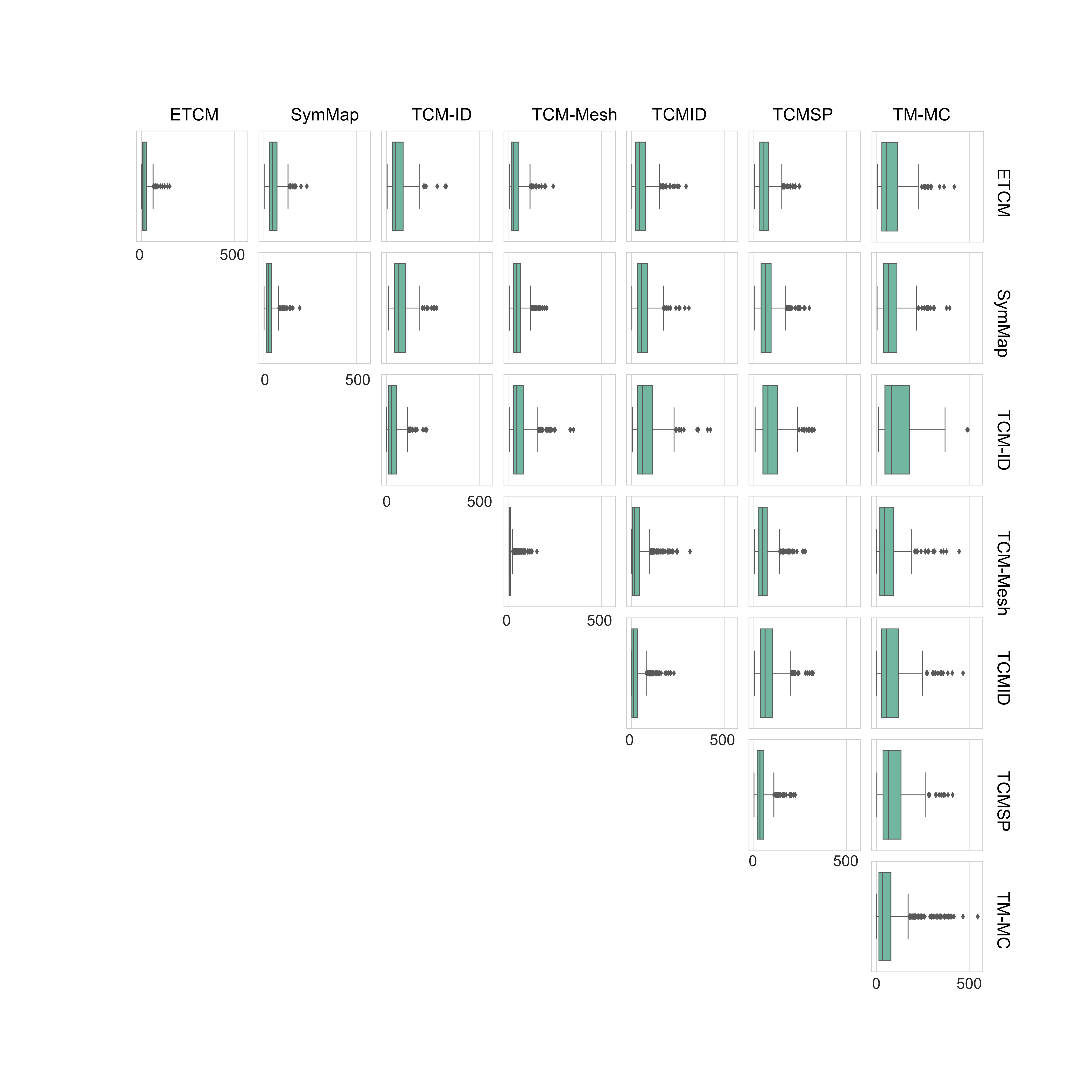

Supplement: Supplementary file 2 [file Image1.png]
